# The role of cytokines and hot flashes in perimenopausal depression

**DOI:** 10.1186/1744-859X-11-9

**Published:** 2012-04-10

**Authors:** Sokratis E Karaoulanis, Alexandros Daponte, Katerina A Rizouli, Andreas A Rizoulis, Georgios A Lialios, Catherine T Theodoridou, Christos Christakopoulos, Nikiforos V Angelopoulos

**Affiliations:** 1 Department of Psychiatry, University Hospital of Larissa, University of Thessaly, Larissa, Greece; 2 Department of Obstetrics and Gynecology, University Hospital of Larissa, Larissa, Greece; 3 Department of Immunology, University Hospital of Larissa, University of Thessaly, Larissa, Greece; 4 Psychiatric Hospital of Attiki, Athens, Greece; 5 Department of Neurology, University Hospital of Odense, Denmark

**Keywords:** antidepressants, cytokines, depression, hot flashes, interleukin 6 (IL-6), interleukin 10 (IL-10), perimenopause, tumor necrosis factor α (TNFα)

## Abstract

**Background:**

An imbalance in the production of proinflammatory and anti-inflammatory cytokines may play a role in the pathophysiology of perimenopausal depression. The aim of this study was to examine serum levels of the proinflammatory cytokines interleukin 6 (IL-6) and tumor necrosis factor α (TNFα), and the anti-inflammatory cytokine IL-10, in perimenopausal women suffering from depression. Furthermore, to assess whether serum cytokine levels are associated with the presence of hot flashes or the use of selective serotonin reuptake inhibitors (SSRIs). We also evaluated the possible association of hot flashes and perimenopausal depression.

**Methods:**

Serum samples from 65 perimenopausal women, 41 with depression and 24 without depression, were assessed for serum IL-6, TNFα and IL-10 by conventional enzyme-linked immunosorbent assays. Depression was evaluated by the 17-item Hamilton Depression Rating Scale (HAM-D 17) and a psychiatric interview. The presence and severity of hot flashes were examined using the Menopause Rating Scale (MRS).

**Results:**

Serum levels cytokines did not differ between depressed women and normal controls. Serum levels of cytokines did not change significantly in depressed women with hot flashes or in depressed women treated with SSRIs. Hot flashes were strongly associated (*P *< 0.0001) with perimenopausal depression.

**Conclusion:**

The study supports the hypothesis that perimenopausal depression is not characterized by increased proinflammatory cytokines and decreased anti-inflammatory cytokines. Women with perimenopausal depression suffer from more severe and more frequent hot flashes than women without perimenopausal depression.

## Introduction

Throughout most of their lives, women are at greater risk of becoming depressed than men. The perimenopause and early postmenopausal period has been considered as 'window of vulnerability', during which emerging physical and emotional discomforts and hormonal changes can lead to the appearance of depression [1-3]. The mechanisms responsible for the development of depression in perimenopausal women remain unclear.

Major depression, in general, is associated with inflammatory processes and neural-immune interactions [4-9]. Proinflammatory cytokines, such as interleukin 6 (IL-6) and tumor necrosis factor α (TNFα) have been considered as key neuromodulators of behavioral, neuroendocrine and neurochemical features of depressive disorders [10]. This view is supported by the frequent appearance of depressive features in cytokine-mediated chronic inflammatory responses, such as rheumatoid arthritis, and in cytokine-treated patients with cancer and chronic hepatitis C [11]. Administration of proinflammatory cytokines in animals induces 'sickness behavior', similar to the behavioral symptoms of depression in humans [8].

Postmenopausal women seem to have higher serum levels of IL-6 and TNFα compared to women of reproductive age [12]. The extent by which the cytokine milieu is altered in women with perimenopausal depression is the focus of ongoing investigation. A single study has reported an association between depression and elevated plasma IL-6 and soluble IL-6 receptor (sIL-6R) [13]. The same study failed to find an association between the levels of these cytokines and the appearance of hot flashes. However, another study showed that serum IL-8 concentrations in premenopausal, perimenopausal and postmenopausal women and bilateral oophorectomized women with severe hot flashes were significantly higher than those in women without hot flashes [14].

Selective serotonin reuptake inhibitors (SSRIs) are a first-line treatment for perimenopausal depression, but hormone therapy could be considered for women experiencing menopausal symptoms unless there are contraindications [15].

The aims of this study were to simultaneously measure the levels of the proinflammatory cytokines IL-6 and TNFα and the anti-inflammatory cytokine IL-10 in women with or without perimenopausal depression, and to assess whether cytokine levels are associated with hot flashes or with the use of selective serotonin reuptake inhibitors. We also examined whether the presence and severity of hot flashes contribute to the presence of perimenopausal depression.

## Methods

### Subjects

A total of 65 women were recruited consecutively as a case series from the Outpatient Clinics and the Departments of Obstetrics & Gynecology and Psychiatry of the University Hospital of Larissa, Greece participated in the study. All participants were Caucasians. Participants were divided into two groups. The first group consisted of perimenopausal women suffering from depression (n = 41) and the second consisted of perimenopausal women without depression (n = 24). All women were in the perimenopausal phase, defined by the presence of irregular cycles or amenorrhea for less than 12 months. The serum levels of follicle-stimulating hormone (FSH) of each of the participant of the study were more than 40 mIU/ml [16].

Exclusion criteria were: the presence of bipolar disorder, other psychiatric diseases (for example, schizophrenia), diseases which can affect the immune system or cause depression (for example, rheumatoid arthritis), the use of medications except for SSRIs, recent stressful situations such as bereavement, hysterectomy, oral contraceptives and hormone replacement therapy.

Among the 41 women with depression, 19 had a history of depression and they were taking SSRIs such as citalopram, fluoxetine or sertraline alone. Each woman had been taking SSRIs for a period of more than a month without any other psychotropic or non-psychotropic medication. These women had experienced more than two episodes of depression in the past and they were still depressed when enrolled into the study (17-item Hamilton Depression Rating Scale (HAM-D 17) score > 10). Consequently, this subgroup of depressed perimenopausal women treated with SSRIs were classed as patients resistant to this class of antidepressants and therefore still depressed. The remaining 22 had their first episode of depression during perimenopause and they had never used psychotropic medications. Depression was diagnosed through a psychiatric interview and administration of the HAM-D 17. A woman was considered depressed if she had scored over 10 on the HAM-D 17 and had fulfilled the criteria of major depression according to the *International Classification of Diseases*, tenth edition (ICD-10). If a woman had scored 10 or less on the HAM-D 17 she was considered normal. Hot flashes were examined using the Menopause Rating Scale (MRS) and they were classified as: absent, mild, moderate, severe or very severe [17,18].

Venous blood samples were collected between 8.00 AM and 12.00 AM. Samples were centrifuged at 3,500 rpm for 5 minutes and aliquots of serum samples were stored at -80°C until further use.

Informed consent for participation in this study was obtained from each woman. The ethics committee of the University Hospital of Larissa approved the study.

### Measurement of serum cytokine concentrations

IL-6, IL-10 and TNFα concentrations were determined by conventional, commercially available ELISA kits (Bender Medsystems, Vienna, Austria) in accordance with the manufacturer's instructions. Each determination was performed in duplicate, and average values were used for statistical analysis. The limits of detection were 0.92 pg/ml for IL-6, 2.3 pg/ml for TNFα and 0.99 pg/ml for IL-10. The intra-assay coefficients of variation were 3.4% for IL-6, 6.0% for TNFα and 3.2% for IL-10. The inter-assay coefficients of variation were 5.2% for IL-6, 7.4% for TNFα and 5.6% for IL-10.

### Statistical analysis

Data analysis was conducted using the commercially available computer software SPSS V.15.0 (SPSS Inc., Chicago, IL, USA). The normality assumption was checked using the Shapiro test. As departures from normality were significant, non-parametric methods were used.

Results are presented as medians (10% to 90%). The differences in cytokine concentrations between depressed and non-depressed perimenopausal women were analyzed with the Mann-Whitney U test. The relationship between hot flashes and depression was analyzed with the χ^2 ^test, and the Kruskal-Wallis test was used in order to detect any association between hot flashes and HAM-D 17 or hot flashes and cytokines. A difference between two groups was considered to be statistically significant when *P *< 0.05.

## Results

The major characteristics of the perimenopausal women with or without depression are summarized in Table 1. The two groups did not differ in terms of age, years of education, months of amenorrhea, smoking habits or marital status. As was expected, depressed women scored higher on the HAM-D 17 than women without depression (Table 1). In many women, the cytokine levels were below the limits of detection [19]. Table 2 shows the number of cases below sensitivity in the clinical sample and in the control sample, respectively.

**Table 1:** Demographic characteristics of women with or without perimenopausal depression

Characteristic	Perimenopausal women with depression (n = 41)	Perimenopausal women without depression (n = 24)	*P *value
Age	50.10 ± 3.95	48.29 ± 10.95	0.94 (NS)^a^
Education, years	8.22 ± 3.87	8.29 ± 3.93	0.94 (NS)^a^
Amenorrhea, months	6.88 ± 4.62	7.10 ± 3.94	0.59 (NS)^a^
Married	36 (87.8%)	22 (91.7%)	0.37 (NS)^b^
Current smokers	15 (36.6%)	7 (29.2%)	0.6 (NS)^b^
HAM-D	16.85 ± 5.02	5.68 ± 2.52	< 0.0001^a^

^a^Mann-Whitney U test.

^b^χ^2 ^test.

HAM-D, Hamilton Depression Rating Scale; NS, not significant.

**Table 2:** Showing the sensitivity threshold for each cytokine and the number of cases below sensitivity in the clinical sample and in the control sample, respectively

Cytokine	Sensitivity threshold (pg/ml)	Perimenopausal women with depression (n = 41); N = below sensitivity (true zeros)	Controls (n = 24); N = below sensitivity (true zeros)
TNFα	2.3	28	18
IL-6	0.92	31	18
IL-10	0.99	32	17

IL = interleukin; TNF = tumor necrosis factor.

### Association of serum cytokine concentrations with perimenopausal depression

The results of serum cytokine concentrations for individual cases of perimenopausal women with or without depression are shown in Table 3. Tables 4 and 5 show the serum concentrations of the considered cytokines in the different subgroups of women (Table 4 shows perimenopausal women with depression not treated and those treated with SSRIs vs perimenopausal women without depression; Table 5 shows the total group of 65 perimenopausal women divided into 5 subgroups on the basis of the severity of hot flashes). The concentration of cytokines of women with depression did not differ significantly from those of normal controls (Table 3). However, it should be noted that for serum levels of TNFα there is an increasing trend and for levels of IL-6 there is a decreasing trend in the two subgroups of perimenopausal women with depression (treated and not treated with SSRIs) when compared to perimenopausal women without depression.

**Table 3:** Serum cytokine concentrations in 41 women with perimenopausal depression, compared to those women without perimenopausal depression (n = 24)

Cytokine	Perimenopausal women with depression (n = 41)	Perimenopausal women without depression (n = 24)	*P *value, Mann-Whitney
TNFα	7.07 (1.79 to 18.10)	3.99 (3.01 to 16.50)	0.537, NS
IL-6	1.89 (0.23 to 4.33)	6.23 (0.95 to 22.10)	0.900, NS
IL-10	3.44 (1.08 to 12.00)	3.18 (1.31 to 12.90)	0.726, NS

The results of cytokines are expressed as medians (10% to 90%). Unit of serum cytokine concentration is pg/ml.

IL = interleukin; NS = not significant; TNF = tumor necrosis factor.

**Table 4:** Serum cytokine concentrations in 41 women with perimenopausal depression, subdivided into those receiving (n = 19) or not (n = 22) selective serotonin reuptake inhibitors (SSRIs) compared to those women without perimenopausal depression (n = 24)

Cytokine	Perimenopausal women with depression		Perimenopausal women without depression, N = 24	*P *value, Kruskal-Wallis
			
	Not treated with SSRIs, N = 22	Treated with SSRIs, N = 19		
TNFα	9.84 (2.3 to 21.90)	6.31 (4.08 to 9.23)	6.66 (3.01 to 16.50)	0.215, NS
IL-6	1.75 (1.13 to 2.95)	2.47 (1.59 to 4.67)	0.00 (0.00 to 5.63)	0.364, NS
IL-10	5.69 (3.39 to 12.00)	3.91 (1.08 to 10.20)	5.46 (1.31 to 12.90)	0.942, NS

The results of cytokines are expressed as medians (10% to 90%). Unit of serum cytokine concentration is pg/ml.

IL = interleukin; NS = not significant; TNF = tumor necrosis factor.

**Table 5:** Serum cytokine concentrations in 65 perimenopausal women subdivided into five groups according to the presence and severity of hot flashes (Menopause Rating Scale)

Cytokine	Hot flashes					*P *value, Kruskal-Wallis
		
	Without (n = 8)	Mild (n = 16)	Moderate (n = 11)	Severe (n = 20)	Very severe (n = 10)	
TNFα	8.67 (3.84 to 13.50)	4.47 (1.84 to 7.35)	5.42 (3.49 to 7.35)	5.82 (4.08 to 9.23)	11.15 (3.01 to 21.90)	0.495, NS
IL-6	22.10	1.19 (0.95 to 1.44)	2.42 (1.13 to 4.31)	3.02 (1.26 to 6.96)	2.11 (1.50 to 2.95)	0.456, NS
IL-10	8.43 (3.97 to 12.90)	1.91	6.57 (2.40 to 12.60)	3.48 (1.08 to 10.20)	5.45 1.31 to 12.00)	0.332, NS

The results of cytokines are expressed as medians (10% to 90%). Unit of serum cytokine concentration is pg/ml.

IL = interleukin; NS = not significant; TNF = tumor necrosis factor.

### Association of serum cytokine concentrations with hot flashes

**Figure 1:**
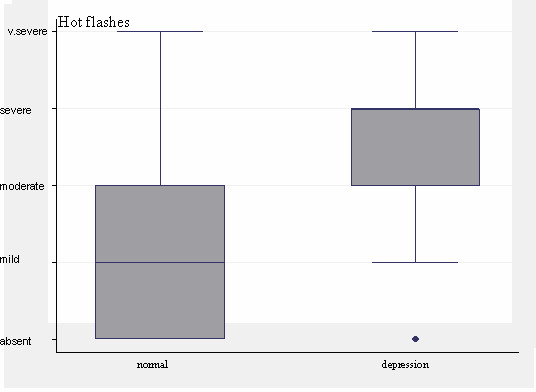
**Perimenopausal women were divided into five groups according to the presence and severity of hot flashes (Menopause Rating Scale)**. The presence and severity of hot flashes were strongly and positively correlated with perimenopausal depression (*P *< 0.0001).

The presence and severity of hot flashes were strongly and positively associated with perimenopausal depression (χ^2 ^test, *P *< 0.0001) (Figure 1). The same strong positive association was detected between hot flashes and the HAM-D 17 score (Kruskal-Wallis, *P *< 0.0001). Depressed women show more hot flashes of the moderate-to-severe type in contrast to the absent-to-moderate type of hot flashes in normal controls.

Perimenopausal women were divided into five groups according to the presence and severity of hot flashes (Menopause Rating Scale). The first group consisted of women with no hot flashes, the second women with mild hot flashes, the third women with moderate hot flashes, the fourth women with severe and the fifth women with very severe hot flashes. The levels of cytokines in these five groups are shown in Table 5. The levels of TNFα (*P *= 0.495), IL-6 (*P *= 0.456) and IL-10 (*P *= 0.332) did not show any statistically significant differences between groups of perimenopausal women subdivided in accordance to the severity of hot flashes.

Similarly, a lack of statistically significant difference was obtained when analysis was carried out in perimenopausal women with depression divided into five groups according to the presence and severity of hot flashes in relation to the levels of TNFα (*P *= 0.583), IL-6 (*P *= 0.719) and IL-10 (*P *= 0.745) in these five groups (Table 6). Moreover, a comparison of the presence and severity of hot flashes was made between depressed women taking SSRIs and depressed perimenopausal women not taking SSRIs. The result was that SSRIs did not affect the presence and severity of hot flashes (Mann-Whitney U, *P *= 0.710).

**Table 6:** Serum cytokine concentrations in 41 perimenopausal women with depression subdivided into five groups according to the presence and severity of hot flashes (Menopause Rating Scale)

Cytokine	Hot flashes					*P *value, Kruskal-Wallis
		
	Without, (n = 2)	Mild, (n = 7)	Moderate, (n = 6)	Severe, (n = 17)	Very severe, (n = 9)	
TNFα	0.00 (true zero)	4.61 (1.84 to 7.35)	7.35	8.99 (4.65 to 14.30)	13.19 (7.12 to 21.90)	0.583, NS
IL-6	0.00 (true zero)	1.44 (1.44 to 1.44)	1.13	2.50 (1.44 to 4.67)	2.11 (1.50 to 2.95)	0.719, NS
IL-10	0.00 (true zero)	0.00 (true zero)	4.54	4.96 (3.10 to 10.20)	6.83 (3.44 to 12.00)	0.745, NS

The results of cytokines are expressed as medians (10% to 90%). Unit of serum cytokine concentration is pg/ml.

IL = interleukin; NS = not significant; TNF = tumor necrosis factor.

## Discussion

The main finding of this study in Caucasian patients is that the proinflammatory cytokines IL-6 and TNFα are not significantly increased and the anti-inflammatory cytokine IL-10 is not decreased in perimenopausal women with depression. This finding does not add strength to the cytokine hypothesis of depression, which supports the view that depression in general is characterized by increased levels of proinflammatory cytokines, such as IL-6 and TNFα, and decreased levels of anti-inflammatory cytokines, such as IL-10. According to this hypothesis specific alterations of the cytokine environment are induced by external and internal stressors and by an increased translocation of lipopolysaccharide (LPS) from Gram-negative bacteria (leaky gut) [20,21]. The study of Ushiroyama *et al*. [13] measured IL-6 in a large sample of non-Caucasian patients. He found increased plasma levels of IL-6 in the subgroup of women with depression and hot flashes when compared to women with hot flashes without depression and to control subjects. However, it has to be mentioned that in our study there was a trend in increasing levels for TNFα and a trend in decreasing levels for IL-6 in perimenopausal women with depression when compared to perimenopausal women without depression. This is in line with the data existing in the literature on the topic as far as TNFα, while it is in contrast to what has been shown for IL-6; it is reported that both cytokines increase significantly in depressed patients when compared to normal subjects [9,22-24].

Our findings are not unexpected as perimenopause is associated with spontaneous increases of proinflammatory cytokines, especially IL-6 and TNFα, attributed to decreased levels of estrogens. This release seems to affect perimenopausal women without distinction among those with or without depression. The mechanism by which estrogens alter cytokine expression is elusive and may include interactions of the estrogen receptors with other transcription factors, modulation of nitric oxide activity, antioxidative effects, plasma membrane actions and changes in immune cell function [25]. According to our findings these cytokine levels are not statistically different among those with or without depression. Circulating cytokine levels may not reflect the local production of cytokines in women with depression and a better understanding of local cytokine levels would be more informative. Cerebrospinal fluid levels of cytokines have been measured in other infectious and non-infectious diseases, including psychiatric disorders such as schizophrenia, as an indicator of central nervous system (CNS) cytokine levels. It is well known that cytokines are synthesized mainly by monocytes, but they can also be secreted by other immune and non-immune cells, such as microglia and astrocytes in the CNS [26].

The design of the present study did not allow for invasive procedures, such as collection of cerebrospinal fluid (CSF) and measurements of cytokine levels in such biological material.

It also needs to be noted that variations in standards and pairs of antibodies used by different companies may affect the validity and the performance of the ELISA kits used for the measurement. Thus, differences in the sensitivity and specificity of these kits have been reported [27].

We found a significant association between hot flashes and perimenopausal depression. Such an association adds support to the 'domino theory' of the pathophysiology of perimenopausal depression. This theory asserts that experience of the somatic symptoms, caused by diminishing estrogen, is the direct cause of the onset of depression during perimenopause. Mental and physical discomforts such as hot flashes, inability to concentrate, insomnia, and vasomotor symptoms, which characterize the menopausal transition, may easily disrupt a woman's emotional life. In particular, when combined with a lack of understanding of or control over their source, these symptoms may lead a woman into a downward spiral towards depression [3,28-33]. The association of hot flashes with perimenopausal depression is not a consistent finding, as Ozturk *et al*. were unable to report it [34].

When we analyzed our data in relation to the administration or not of SSRIs, we found that despite their presumed immunomodulatory properties (in favor of decreased IL-6) [35,36], SSRIs are not linked to any specific cytokine. Suppression of proinflammatory cytokines does not occur in patients who fail to respond to SSRIs (treatment-resistant depression) [37] and our report demonstrates that this is also the case in perimenopausal depression. A plausible explanation for this would be that SSRIs cannot relieve women from severe vasomotor and other symptoms associated with decreased estrogen, which cause the perimenopausal depression; consequently, perimenopausal depression becomes refractory to treatment with antidepressants and proinflammatory cytokines are not suppressed in depressed perimenopausal women who take SSRIs.

Cytokines such as IL-6 and TNFα appear to be increased in menopause and to be potent vasodilators [38,39]. This raises the question as to whether they would be further increased in the subgroup that experienced hot flashes. It appears that in our enrolled perimenopausal women neither IL-6 and TNFα or IL-10 were associated with the presence or the severity of hot flashes.

The present study has several limitations. First of all circulating cytokine levels do not necessarily reflect the local production of cytokines in women with depression. Moreover, this study is a preliminary report and its data should be confirmed by studies that include larger samples of patients and controls.

## Conclusions

It seems that hot flashes play a crucial role in the presence of perimenopausal depression. The inflammatory theory of depression is not suitable for perimenopausal depression, since serum concentrations of the proinflammatory cytokines TNFα and IL-6 are not raised. Perimenopausal depression has unique characteristics that must be taken into account during its treatment, with an emphasis on hormone replacement therapy that will alleviate the hot flashes and improve the perimenopausal depression prognosis.

## Competing interests

The authors declare that they have no competing interests.

## Authors' contributions

SEK: design, interpretation, preparation of manuscript, editing, revising. AD: recruitment, preparation of manuscript, revising. KAR: carried out the immunoassays. AAR: data collection. GAL: recruitment. CTT: carried out the immunoassays. CC: design, interpretation. NVA: conception of the study, overall coordination.

All authors read and approved the final manuscript.
